# Evaluating the suitability of six hydrophobic natural eutectic solvents as the internal phase in a cosmetic cream formulation

**DOI:** 10.1111/ics.70097

**Published:** 2026-03-29

**Authors:** A. Verger, R. Grard, E. Vrignaud, X. Perse, S. Bouderbala, L. Boudesocque‐Delaye, M. Grisel, E. Gore, E. Munnier

**Affiliations:** ^1^ Université de Tours, UPR CNRS 4301 CBM, département NMNS, Faculté de Pharmacie Tours France; ^2^ Université Le Havre Normandie, Normandie Univ, URCOM UR 3221 Le Havre France; ^3^ Université de Tours, UR 7502 SIMBA, Faculté de Pharmacie Tours France

**Keywords:** cream, emulsion, hydrophobic natural eutectic solvents, in vitro sensory approach, stability

## Abstract

**Objective:**

This work evaluates whether hydrophobic natural eutectic solvents can replace the conventional oil phase in cosmetic creams while maintaining or improving physicochemical stability, rheological structure, and sensory performance. The goal is to develop formulations that combine sustainability, regulatory compliance, and sensory performance while remaining compatible with standard low‐energy manufacturing processes.

**Methods:**

Six HNESs systems composed of regulation‐compliant fatty acids, menthol, and alkane diols were characterized for viscosity, density, and refractive index. Model oil‐in‐water creams were prepared using cold homogenization to ensure scalability and energy efficiency. Emulsion stability was assessed over 30 days in accelerated ageing conditions using multiple light scattering and pH tracking; rheology was evaluated via flow curves and strain sweep tests; applicative sensory properties (spreading resistance, gloss, stickiness, residual film wettability) were instrumentally measured on a non‐biological skin surrogate.

**Results:**

Solvent composition governed emulsion behaviour. Fatty acid‐based systems produced small, stable droplets, low bulk viscosity, and favourable sensory attributes (high lubrication, reduced stickiness), while maintaining dermatologically acceptable pH. Menthol‐containing systems exhibited higher viscosities and strong gel‐like networks but showed physical instability under storage. Alkane diol‐rich systems improved lubrication and gloss yet displayed larger droplet growth and pH drift, indicating weaker long‐term stability. Across all creams, residual films decreased the water contact angle markedly, evidencing hydrophilic surface behaviour after application, and immediate gloss increased versus triglycerides control.

**Conclusion:**

HNESs can function as structural oily phase substitutes in creams prepared by low‐energy processes, enabling sustainable, regulation‐compliant formulations with desirable sensory profiles. Fatty acid‐based systems offer the most robust balance of stability and user experience, whereas menthol‐ and alkane diol‐rich systems require compositional optimization to mitigate storage instability while preserving sensory benefits.

## Graphical abstract

Hydrophobic natural eutectic solvents can function as structural oil phase substitutes in creams prepared by low‐energy processes, enabling sustainable, regulation‐compliant formulations with desirable sensory profiles.

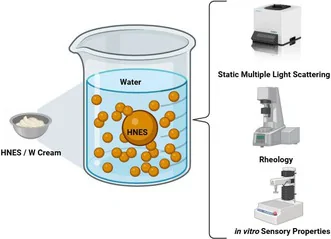

## RÉSUMÉ

**Objectif:**

Ce travail évalue si les solvants eutectiques naturels hydrophobes peuvent remplacer la phase huileuse conventionnelle des crèmes cosmétiques tout en maintenant ou en améliorant la stabilité physico‐chimique, la structure rhéologique et les performances sensorielles. L’objectif est de développer des formulations qui combinent durabilité, conformité réglementaire et performance sensorielle tout en restant compatibles avec les processus de fabrication standard à faible énergie.

**Méthodes:**

Six systèmes de solvants eutectiques naturels hydrophobes (SENH) composés d’acides gras, de menthol et de diols alcaniques conformes à la réglementation ont été caractérisés en termes de viscosité, de densité et d’indice de réfraction. Des crèmes modèles huile‐dans‐eau ont été préparées en utilisant l’homogénéisation à froid pour assurer l’évolutivité et l’efficacité énergétique. La stabilité de l’émulsion a été évaluée sur 30 jours dans des conditions de vieillissement accéléré à l’aide de la diffusion multiple de la lumière et du suivi du pH ; la rhéologie a été évaluée par des courbes de débit et des tests de balayage de déformation ; les propriétés sensorielles applicatives (résistance à l’étalement, brillance, caractère collant, mouillabilité du film résiduel) ont été mesurées instrumentalement sur un substitut cutané non biologique.

**Résultats:**

Le comportement de l’émulsion était régi par la composition du solvant. Les systèmes à base d’acides gras (par ex., acide octanoïque/laurique) ont produit de petites gouttelettes stables, une faible viscosité en vrac et des attributs sensoriels favorables (lubrification élevée, caractère collant réduit), tout en maintenant un pH dermatologiquement acceptable. Les systèmes contenant du menthol présentaient des viscosités plus élevées et de forts réseaux de type gel, mais présentaient une instabilité physique au stockage. Les systèmes riches en diols alcaniques ont amélioré la lubrification et la brillance, mais ont affiché une croissance plus importante des gouttelettes et une dérive du pH, indiquant une plus faible stabilité à long terme. Sur l’ensemble des crèmes, les films résiduels ont nettement réduit l’angle de contact avec l’eau, attestant d’un comportement de surface hydrophile après l’application, et la brillance immédiate a augmenté par rapport au témoin aux triglycérides.

**Conclusion:**

Les solvants eutectiques naturels hydrophobes peuvent fonctionner comme des substituts structurels de la phase huileuse dans des crèmes préparées par des processus à faible énergie, permettant des formulations durables et conformes à la réglementation avec des profils sensoriels souhaitables. Les systèmes à base d’acides gras offrent l’équilibre le plus robuste entre la stabilité et l’expérience d’utilisation, tandis que les systèmes riches en menthol et en diols alcaniques nécessitent une optimisation de la composition pour atténuer l’instabilité au stockage tout en préservant les avantages sensoriels.

## INTRODUCTION

The cosmetic industry is undergoing a significant transformation driven by increasing consumer demand for safer, more sustainable, and environmentally responsible products. This shift aligns with a broader societal movement toward ecological consciousness, where consumers are no longer solely motivated by product performance but are equally attentive to ingredient origin, biodegradability, ethical sourcing, and overall environmental footprint [1]. In response, cosmetic formulators are under growing pressure to re‐evaluate traditional ingredients and explore innovative, bio‐based alternatives that uphold both efficacy and sustainability. Among the components under scrutiny, the hydrophobic oily phase of cosmetic emulsions—typically composed of mineral oils, synthetic esters, or silicones—has drawn significant attention. Although these ingredients confer desirable sensory and rheological properties, their petrochemical origin, low biodegradability, and potential for bioaccumulation present substantial drawbacks. Replacing conventional materials with biocompatible, renewable alternatives remains a central challenge for modern green formulation strategies [2, 3]. Vegetable oils are among the primary candidates for replacing controversial substances in the cosmetic emulsions' oily phase. Their inherent physicochemical properties and natural compatibility with the skin position them as highly valuable ingredients. Nevertheless, sustainable sourcing practices are imperative to minimize environmental impact. Additionally, their susceptibility to oxidative degradation, seasonal variability in chemical composition, occasional heaviness in sensory attributes, and potential allergenicity impose significant formulation challenges [4]. Finally, considering their cost, these oils are typically incorporated at low concentrations within the oily phase of emulsions combined with diverse emollients or medium/long‐chain triglycerides obtained by synthesis, hemisynthesis, or biotechnology methods [5]. These challenges underscore the need for innovative oily phase substitutes that combine sustainability with functional performance.

Natural eutectic solvents (NES) have recently emerged as promising candidates. Formed by mixing natural compounds, such as fatty acids, polyols, or amino acids in specific ratios, NES exhibit melting point depression, resulting in stable liquids at room temperature. When this depression exceeds 10 K, they are classified as Natural Deep Eutectic Solvents (NaDES) [6]. Unlike traditional organic solvents, NES are typically non‐volatile, biodegradable, non‐toxic, and derived from renewable feedstocks, making them highly compatible with the principles of green chemistry. The unique tunability of NES composition allows the design of solvents with tailored polarity ranging from hydrophilic to amphiphilic or hydrophobic systems [7, 8, 9]. Their ability to dissolve a wide range of active compounds, including both polar and non‐polar molecules, has been extensively documented, particularly in pharmaceutical and natural product extraction fields. Until now, hydrophobic natural eutectic solvents (HNES), mainly based on terpenoids and medium‐chain fatty acids, have been used for their solvent properties in the fields of water purification [10, 11], green synthesis [12], green analysis [13], and extraction of hydrophobic molecules of interest from diverse biomasses [14, 15]. In the fields of skin care and health, they were mainly used as extraction solvents for free fatty acids [16] or carotenoids [17]. Moreover, some of them were demonstrated to have intrinsic wound healing and antibacterial properties [18].

Despite this growing body of literature, the direct incorporation of HNES into cosmetic formulations as structural components replacing conventional emollients remains largely unexplored. This study investigates the feasibility of replacing the conventional hydrophobic phase of cosmetic creams with HNES. Six HNES systems based on regulation‐compliant ingredients were evaluated for their ability to provide structural, rheological, and in vitro sensory properties comparable to traditional oils: octanoic acid/lauric acid (4:1, mol/mol), octanoic acid/1,3‐propanediol (5:1, mol/mol), octanoic acid/1,3‐propanediol (1:2, mol/mol), octanoic acid/menthol (1:1, mol/mol), lauric acid/menthol (1:2, mol/mol), and menthol/1,2‐octanediol (1:1, mol/mol). By identifying the strengths and limitations of each system, this work aims to establish a foundation for eco‐designed, high‐performance cosmetic emulsified products.

## MATERIALS AND METHODS

### Chemicals

Glycerin, 99%, lauric acid (C12), 99%, octanoic acid (C8), 99%, 1,2‐octanediol, 98% and 1–3, propanediol, 98% were purchased from Thermo Scientific (Waltham, USA). Xanthan gum and L‐menthol were obtained from COOPER (Melun, France). Amigum® (sclerotium gum), was a kind gift from Alban Muller (Fontenay‐sur‐Eure, France). The Lipoid P75‐3®, hydrogenated lecithin, was a kind gift from Lipoid Kosmetik (Ludwigshafen, Germany). Benzyl alcohol and dehydroacetic acid were obtained from Fisher Scientific (Illkirch, France). Water was purified using a Milli‐Q system (Millipore Corporation, Bedford, MA, USA).

### Hydrophobic NES formulation

HNES C8/C12 (4:1, mol/mol) [19, 20], C8/1,3‐Propanediol (1:2, mol/mol) [21], C8/1,3‐Propanediol (5:1, mol/mol) [16], C8/Menthol (1:1, mol/mol) [10, 15, 22], Menthol/1,2‐Octanediol (1:1, mol/mol) [23, 24] and C12/Menthol (1:2, mol/mol) [25, 26] were prepared by mixing components together. The mixture was heated at 50°C and stirred by magnetic stirring for 1 h until a colourless liquid was obtained. After returning to room temperature, HNES are stored for a maximum of 7 days at 20°C in an oven. Results from various analyses of HNES, including shear‐dependent viscosity at 25°C using a Kinexus pro+ rheometer (Netzsch) with a sandblasted Couette geometry.

### Formulation and preparation of creams

A basic cream suitable for COSMOS (COSMetic Organic and Natural Standard) certification was chosen as the formulation chassis to evaluate the ability to incorporate a hydrophobic NES. The detailed composition is presented in Table 1.

**TABLE 1:** Quantitative composition of the creams.

Ingredients	Function	Contents in creams (% Wt.)
Sclerotium gum	Thickener	0.75
Xanthan gum	Thickener	0.25
Glycerol	Humectant	10
Hydrophobic natural eutectic solvent	Solvent	25
Hydrogenated lecithin	Emulsifier	3
Dehydroacetic acid/ benzyl alcohol mix	Antimicrobial agent	1
Water	Solvent	q.s 100

Creams were prepared at room temperature using a Hauschild SpeedMixer DAC 150.1 CM148 (Hauschild, Hamm, Germany). The aqueous phase was first obtained by introducing glycerin, water, and thickeners into the SpeedMixer cup and homogenizing for 4 min at 1800 rpm. Subsequently, the oil phase, comprising the hydrophobic NES with the preservative and emulsifier, was added on top of the aqueous phase in the same vessel and mixed for an additional 2 min at 1800 rpm using the Hauschild pulverizing ring set. Each formulation was prepared in triplicate (*n* = 3). The resulting creams were stored for 30 days in a climatic chamber (Memmert, Büchenbach, Germany) maintained at 40°C and 75% relative humidity. The pH was recorded 24 h (D1) and 30 days (D30) after preparation, following a 20‐min stabilization period before measurement. Formulation codes are indicated in Table 2.

**TABLE 2:** Denomination of the formulations including hydrophobic natural eutectic solvents as the internal phase.

Sample	Code
C8/C12 (4:1, mol/mol)	C_C8C12
C8/1,3‐propanediol (5:1, mol/mol)	C_C8P_5:1_
C8/1,3‐propanediol (1:2, mol/mol)	C_C8P_1:2_
C8/Menthol (1:1, mol/mol)	C_C8M
C12/Menthol (1:2, mol/mol)	C_C12M
Menthol/1,2‐octanediol (1:1, mol/mol)	C_MO

### Static multiple light scattering‐based stability analysis

Static multiple light scattering‐based stability analyses were done with the Turbiscan® instrument (Microtrac, Toulouse, France). The droplet size is also calculated thanks to the software. The analysis was made 24 h after the preparation on each sample in a specific 4 mL vial.

### Rheological characterization

Rheological analyses were performed with a Kinexus pro+ rheometer (Netzsch, Germany) maintained at 25°C, a temperature representative of actual product use and suitable for assessing formulation stability, user comfort, and compliance with cosmetic testing standards. Each measurement was carried out in triplicate, using a fresh sample for every run. Prior to testing, samples were allowed to relax for at least 15 min to ensure thermal and structural equilibration. Flow behaviour was characterized through continuous shear tests employing a sanded cone–plate geometry (4° angle, 40 mm diameter); apparent viscosity was determined under increasing shear rates from 0.1 to 1000 s^−1^ over 150 s. The viscoelastic properties were further evaluated by oscillatory measurements (amplitude sweep—1 Hz, 0.1–100% strain).

### Applicative in vitro sensory properties

To assess sensory‐related properties and recognize those brought by the hydrophilic phase, a control formulation was prepared using the same process by substituting the hydrophobic natural extract (HNES) with an equivalent amount of a common component of the oily phase, medium‐chain triglycerides (MCT).

#### Spreading behaviour during rub‐out

Friction value was assessed using a Frictiometer® FR700 (Courage‐Khazaka electronic GmbH MPA580, Germany), equipped with a smooth, flat Teflon (PTFE) disk of 16 mm in diameter, applied to a Biody® Plate #30. Using a micropipette, 20 μL of product was dispensed onto the 2 cm diameter test area of the plate. The product was then evenly spread by performing five circular motions to ensure uniform layer thickness. Subsequently, the Frictiometer® was positioned on the treated area, and measurements were recorded for 120 s at 90 RPM. Each product was tested for at least three independent measurements to ensure repeatability.

#### Contact angle

The contact angle was assessed on the residual film using a drop shape analyser DSA 30 (Krüss GmbH, Germany). Using a micropipette, 13 μL of the product was deposited on a 4.9 cm diameter circular area on the Biody® Plate #30. The product was spread by performing 10 rotations at 90 RPM. After a 3‐min resting period, a drop of distilled water was deposited on the corresponding surface, and the contact angle between the drop and the product layer on the Biody® surface was measured. Measurements were performed in triplicate for each product.

#### Glossiness

The residual film gloss was evaluated immediately after product application and again 5 min later using a Glossimeter (Courage‐Khazaka electronic GmbH MPA580, Germany) to monitor changes in surface reflectivity over time. A 30 μL sample of the product was accurately applied onto a 4 cm diameter circular area on the Biody® Plate #30 using a micropipette. The product was then uniformly spread by performing 20 rotations at 90 RPM, after which the initial gloss value was measured. A second measurement was recorded 5 min later to assess any variation in gloss over time. Each product was tested for at least three independent measurements.

#### Stickiness

A Biody® Plate #30 artificial skin surface was used to perform the stickiness of the residual film remaining after product rub‐out using a TA.XT Plus Texture Analyser (Stable Micro Systems, UK), equipped with a P/025 spherical stainless steel probe and a 500 g load cell, following the protocol described previously [27]. Using a micropipette, 35 μL of product was accurately dispensed onto a 9.6 cm^2^ circular area of the Biody® Plate #30. The product was evenly spread by applying 10 rotations at 90 RPM and allowed to rest for 3 min prior to measurement. The force versus time curve was recorded, and the area under the curve in g.s, representing the work necessary to detach the probe from the skin surface, was calculated using Texture Exponent software. Each product was tested at least in triplicate.

### Statistical analysis

Results are expressed as mean values ± standard deviation (SD). The Mann–Whitney test was used for statistical analysis using the GraphPad Prism 8.0 software. Viscoelastic modulus results and relaxation times were obtained individually for each replicate using the Rspace software supplied with the rheometer and then averaged using R software. For spreading and residual film properties, statistical analyses were performed using XLSTAT software, version 2016.02.27444 (Addinsoft, France). A one‐way ANOVA was used to assess differences among products. When significant differences were observed (*p* < 0.001), product groupings were established through Tukey's post hoc multiple comparison test.

## RESULTS

### Hydrophobic NES characterization

The physicochemical characterization of the different HNES reveals distinct profiles depending on both the nature and molar ratio of the constituents (Table 3). The viscosities at 25°C range from 0.008 Pa·s (C8/C12) to 0.073 Pa·s (Menthol/1,2‐Octanediol), indicating structural diversity. Interestingly, the menthol‐based HNES, especially Menthol/C12 and Menthol/1,2‐Octanediol, exhibit the highest viscosities, suggesting stronger molecular interactions or more extensive hydrogen bonding networks, potentially due to the presence of rigid, hydrophobic and amphiphilic structures like menthol and long‐chain diols [15]. The density values are also informative. NES based on medium‐chain fatty acids and diols exhibit higher densities, with a maximum of 0.981 for C8/1,3‐Propanediol (1:2). This could reflect tighter molecular packing in the presence of highly polar or small molecular weight components [28]. In contrast, systems rich in menthol and longer chain fatty acids tend to exhibit lower densities (~0.896–0.912), consistent with the bulkier, less dense nature of these components [29]. Refractive index (*n*) varies between 1.430 (C8/C12 and C8/1,3‐Propanediol 5:1) and 1.473 (Menthol/1,2‐Octanediol) and shows a clear trend: higher refractive indices are associated with systems containing menthol and secondary alcohols, such as 1,2‐Octanediol. This may suggest increased polarizability and molecular organization, supporting the idea of significant hydrogen bonding and complex network structures in these systems [30, 31]. Overall, these data indicate that HNES molecular architecture, modulated by the chemical nature and molar ratios of their constituents, has a direct impact on their bulk physicochemical properties. These parameters are likely to influence their behaviour in emulsified formulations. HNES were physically stable for at least 6 months at 20°C.

**TABLE 3:** Hydrophobic natural eutectic solvents viscosity (*η*), density (*d*) and refractive index (*n*) at 25°C.

Sample	*η* (Pa.s)	*d*	*n*
C8/C12 (4:1, mol/mol)	0.008 ± 0.001	0.904 ± 0.081	1.430 ± 0.172
C8/1,3‐propanediol (5:1, mol/mol)	0.010 ± 0.002	0.922 ± 0.083	1.430 ± 0.172
C8/1,3‐propanediol (1:2, mol/mol)	0.028 ± 0.004	0.981 ± 0.088	1.438 ± 0.173
C8/Menthol (1:1, mol/mol)	0.019 ± 0.003	0.904 ± 0.081	1.468 ± 0.176
C12/Menthol (1:2, mol/mol)	0.030 ± 0.005	0.896 ± 0.081	1.472 ± 0.177
Menthol/1,2‐octanediol (1:1, mol/mol)	0.073 ± 0.011	0.912 ± 0.082	1.473 ± 0.177

### Creams pH measurements

The pH values of the creams (Table 4) fall within a range (4.2–5.5) suitable for topical application [32]. However, some HNES‐based creams exhibit notable pH variation over the 30‐day storage period. For example, C_C8P_1:2_ demonstrates a marked drop from pH 4.93 to 4.10. Both C_C8M and C_MO formulations were physically unstable by Day 30, which precluded pH measurements. This instability might be linked to insufficient shearing during emulsification, crystallization phenomena, or incompatibility between the HNES and the emulsifier system. Given their relatively low density and moderate viscosity, it is plausible that these HNES caused phase separation or destabilization via coalescence [33]. In this case, the cream stability could be improved by modifying the nature or concentration of the surfactant and thickening agent and/or by optimizing the manufacturing process.

**TABLE 4:** Creams pH measurements at Day 1 and Day 30.

Sample	pH (D1)	pH (D30)
C_C8C12	4.56 ± 0.02	5.13 ± 0.09
C_C8P_5:1_	4.27 ± 0.08	4.21 ± 0.13
C_C8P_1:2_	4.93 ± 0.03	4.10 ± 0.13
C_C8M	4.71 ± 0.01	NA
C_C12M	5.35 ± 0.02	5.39 ± 0.12
C_MO	5.21 ± 0.07	NA

*Note*: Data represent the mean ± SD.

Abbreviation: NA, non‐applicable.

### Static multiple light scattering‐based stability analysis

Static multiple light scattering results (Table 5) provide insight into the droplet size evolution over time, which serves as an indirect measure of emulsion stability. Most HNES‐based formulations exhibited an increase in droplet diameter between Day 1 and Day 30, indicative of moderate to severe destabilization processes such as coalescence or Ostwald ripening. Among the stable formulations, C_C8C12 had the smallest and most stable droplet size profile (2.2 μm to 2.9 μm), which correlates well with its low viscosity and low density [33]. In contrast, formulations like C_C8P_1:2_ and C_C12M showed significant increases in droplet size (24.0 μm and 19.5 μm, respectively), pointing to poorer interfacial compatibility or insufficient stabilization over time. The most unstable were C_C8M and C_MO, which could not be assessed at Day 30 due to physical instability. These two systems share notable physicochemical traits: both contain menthol and have high viscosities and refractive indices compared with the other HNES. Such characteristics might promote crystallization or aggregation at the interface, disrupting emulsion integrity [34, 35]. These observations suggest that high viscosity and refractive index in the HNES phase may negatively correlate with emulsion stability, potentially due to poor diffusion into the continuous phase and incompatibility with surfactants.

**TABLE 5:** Creams droplets diameter Day 1 and Day 30.

Sample	Diameter D1 (μm)	Diameter D30 (μm)
C_C8C12	2.2 ± 0.0	2.9 ± 0.5
C_C8P_5:1_	9.0 ± 0.4	11.2 ± 0.5
C_C8P_1:2_	19.2 ± 0.7	24.0 ± 0.9
C_C8M	10.3 ± 0.5	NA
C_C12M	15.6 ± 0.2	19.5 ± 0.3
C_MO	11.9 ± 0.6	NA

*Note*: Data represent the mean ± SD.

Abbreviation: NA, non‐applicable.

### Rheology

Figure 1 illustrates the flow curves of the creams formulated with different HNES, highlighting their shear‐thinning behaviour. Across all samples, viscosity (𝜂) decreases consistently with increasing shear rate, typical of pseudoplastic fluids, and confirming the non‐Newtonian nature of these systems. At low shear rates (0.1 s^−1^), the C_C12M formulation exhibits the highest viscosity values (>215 Pa·s), suggesting well‐structured internal networks, likely due to a high degree of droplet packing and/or strong inter‐droplet interactions (Table 6). On the contrary, C_C8P_1:2_ displays the lowest viscosity (29.9 Pa·s), correlating with its superior physical stability and small droplet size. This suggests that a less viscous, more mobile internal structure may favour emulsion stability by facilitating better droplet dispersion and avoiding bridging or aggregation phenomena. Interestingly, the two formulations that are unstable by Day 30, C_C8M and C_MO show intermediate viscosities (102.8 and 104.9 Pa·s at 0.1 s^−1^) that drop rapidly with increasing shear rate. Their moderate initial resistance to deformation, followed by sharp structural breakdown under stress, may reflect a fragile internal network, potentially explaining their poor physical stability.

**FIGURE 1:**
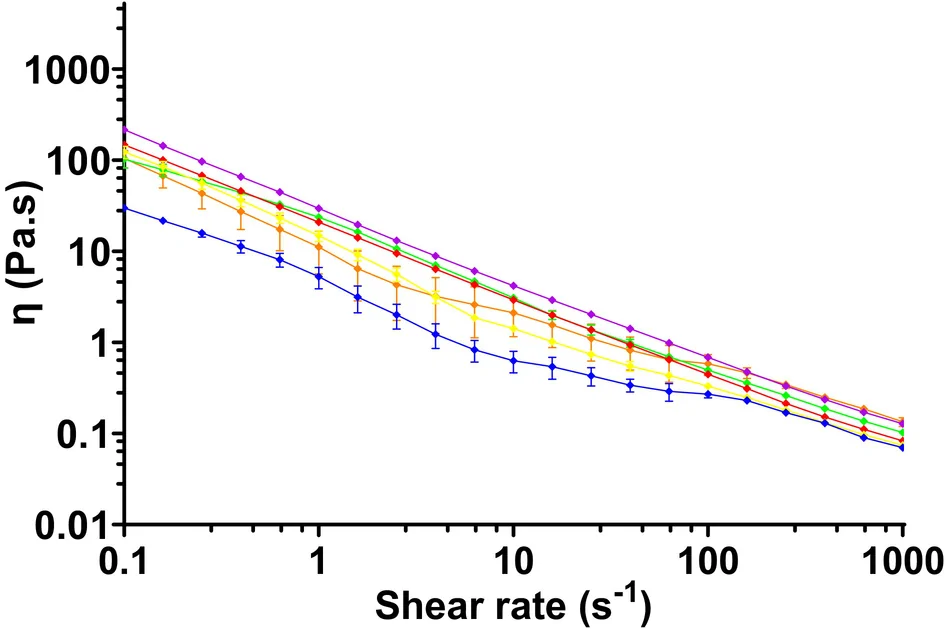
Flow properties of creams C_C8C12 (blue), C_C8P_5:1_ (yellow), C_C8P_1:2_ (red), C_C12M (violet) C_C8M (green), C_MO (orange).

**TABLE 6:** Value of viscosity (in Pa·s) obtained at different shear rates (in s^−1^) during the flow test of creams studied.

Samples	*η* (0.1)	*η* (1)	*η* (10)	*η* (100)	*η* (1000)
C_C8C12	29.90 ± 0.02	5.26 ± 1.39	0.63 ± 0.17	0.27 ± 0.02	0.07 ± 0.01
C_C8P_5:1_	122.90 ± 18.69	14.77 ± 2.01	1.42 ± 1.39	0.28 ± 0.11	0.07 ± 0.01
C_C8P_1:2_	147.23 ± 4.90	20.93 ± 0.95	2.93 ± 0.09	0.45 ± 0.01	0.08 ± 0.01
C_C8M	102.76 ± 20.97	23.61 ± 1.11	3.09 ± 0.12	0.50 ± 0.03	0.10 ± 0.01
C_C12M	215.63 ± 4.56	29.50 ± 0.66	4.18 ± 0.14	0.68 ± 0.02	0.13 ± 0.01
C_MO	104.90 ± 22.58	11.17 ± 5.51	2.11 ± 0.96	0.58 ± 0.15	0.13 ± 0.01

A clear relationship can be observed between the intrinsic viscosity of the HNES (as described in Section 3.1) and the viscosity of the corresponding cream formulations. Systems incorporating more viscous HNES, such as C12/Menthol (0.030 Pa·s), produced creams with higher viscosities (C_C12M), whereas those containing less viscous HNES, such as C8/C12 (0.008 Pa·s), resulted in less viscous creams (C_C8C12).

This trend indicates that the HNES plays a key role in structuring the internal phase of the emulsion, likely by influencing droplet–droplet interactions or through direct association with the emulsifier network. Furthermore, formulations containing menthol and long‐chain diols consistently exhibited increased viscosity, which could be attributed to steric ordering induced by menthol molecules, hydrogen bonding between 1,2‐octanediol and the surrounding matrix that enhances network elasticity, or the limited miscibility of these compounds with the aqueous phase, which increases internal resistance. This rheological behaviour also aligns with the observed instability of the MC8 and MO‐based formulations, despite their relatively high viscosities, the internal structure may lack sufficient robustness under storage conditions, resulting in phase separation or creaming.

Strain sweep tests (Table 7) reveal that for all formulations, the elastic modulus (G') dominates over the viscous modulus (G"), confirming a gel‐like structure with solid‐like behaviour in the linear viscoelastic region (LVR) [6]. This is a typical signature of well‐structured emulsions, where interfacial and network interactions dominate [36]. Among the samples, C_C12M exhibits the highest flow threshold (32.7 Pa), indicating a strong internal structure resistant to deformation.

**TABLE 7:** Rheological data (means ± SD) obtained from the strain sweep tests (NA: non‐applicable).

Samples	Linear viscoelastic region (LVR) (Pa)	Flow threshold (Pa)
C_C8C12	1.97 ± 0.51	15.75 ± 1.19
C_C8P_5:1_	3.99 ± 0.56	23.83 ± 1.32
C_C8P_1:2_	4.65 ± 0.72	NA
C_C8M	1.71 ± 0.77	23.65 ± 1.39
C_C12M	4.67 ± 0.67	32.71 ± 1.27
C_MO	1.71 ± 0.77	23.65 ± 1.39

A clear trend emerges from these physicochemical analyses: low‐viscosity HNES such as C8/C12 (0.008 Pa·s) tend to produce emulsions with smaller droplet sizes, lower overall viscosity, and superior stability at Day 30. In contrast, high‐viscosity NES, especially those involving menthol and diols (e.g., Menthol/1,2‐octanediol, 0.073 Pa·s), result in highly structured emulsions, with elevated elastic moduli and flow thresholds, but in some cases, limited long‐term stability. These findings imply that an overly rigid internal structure does not necessarily confer superior stability, and may, in fact, be detrimental if it lacks adaptability under storage conditions. Moreover, formulations based on simpler or more apolar NES, such as C8/C12 or C8/1,3‐propanediol (5:1), show greater stability despite lower viscosity and mechanical strength. This may reflect better compatibility with the oil phase, more effective distribution within droplets, and greater molecular mobility, which allows the emulsion to dynamically adjust to small perturbations. Finally, it is worth noting that all creams exhibited G′ > G″, confirming the presence of structured, elastic networks. This is beneficial from a sensorial standpoint (e.g., perceived creaminess and spreadability), but not necessarily indicative of long‐term stability.

### Applicative in vitro sensory properties

Evaluation of the sensory‐related properties of the creams was assessed using in vitro instrumental physicochemical methods. The applicative properties were assessed on non‐biological skin Biody® Plate #30 to evaluate resistance as initial haptic perception, reflecting how easily the formulation can be applied on skin and how it behaves once applied in terms of glossiness and stickiness [37, 38, 39]. Results presented in Table 8 clearly indicate that all the creams exhibit lubricating properties. The friction force required to spread the tested formulations is lower than that of the control cream, with statistically significant differences observed for C_C8C12, C_C8P_5:1_, C_C12M. These results suggest that the HNES employed exhibit superior lubricating properties compared with caprylic/capric triglycerides. In contrast, the C_C8P_1:2_ formulation does not differ significantly from the control, indicating that the C8/1,3‐propanediol (1:2) mixture exhibits similar lubricating behaviour to the reference emollient. Notably, when the same HNES (C8/1,3‐propanediol) is used at a higher ratio, an enhancement in spreading performance is observed, attributable to improved lubrication. These findings suggest that a higher proportion of C8 relative to 1,3‐propanediol may enhance lubricating properties. C8 therefore seems to drive the lubricating properties [21].

**TABLE 8:** Means value and standard deviation for circular spreading value, contact angle, and stickiness.

Samples	Circular spreading friction value (AU)	Contact angle (°)	Stickiness (g.s)
Blank (Biody®)	226 ± 27^a^	124 ± 2^a^	0.003 ± 0.001^a^
Control	92 ± 7^b^	26 ± 3^b^	0.491 ± 0.048^e^
C_C8C12	76 ± 8^d^	12 ± 0^c^	0.157 ± 0.025^c^
C_C8P_5:1_	73 ± 7^d^	9 ± 1^c^	0.142 ± 0.006^bc^
C_C8P_1:2_	87 ± 11^bc^	13 ± 2^c^	0.083 ± 0.009^b^
C_C12M	81 ± 7^cd^	11 ± 3^c^	0.320 ± 0.016^d^

*Note*: a–e Means sharing common superscripts are non‐significantly different when tested by Tukey's test (*p* > 0.01).

Biody® exhibits negligible stickiness (0.003 ± 0.001 g·s), comparable to that of human skin, which is naturally non‐sticky [21]. Table 8 indicates that all creams leave a residual adhesive film after application, with varying degrees of stickiness. The control cream, formulated with MCT, shows the highest stickiness (0.491 ± 0.048 g·s), suggesting a strong film‐forming capacity but also a greater tendency to leave a sticky residue. In contrast, all HNES‐based formulations display significantly lower stickiness values, indicating that the incorporation of NES contributes to improved sensory comfort post‐application.

Table 8 also presents the contact angle measurements between a water droplet and the product‐coated surface, taken 3 min after application on the Biody® plate, an approach supported by literature reporting excellent correlations between in vivo measurements and artificial skin substrates [40]. The Biody® surface exhibits a strongly hydrophobic character characterized by a high initial contact angle (124 ± 2°). Upon application of the creams, a significant and consistent decrease in contact angle is observed for all formulations, reaching values as low as 9 ± 1°. This drastic reduction reflects a substantial increase in surface wettability, indicating that the residual films left by the creams confer a pronounced hydrophilic character. Although Biody® plates provide a reproducible and well‐controlled non‐biological surface, they do not reproduce the dynamic hydration, absorption, and lipid reorganization processes occurring on human skin. Consequently, contact angle measurements performed shortly after application mainly reflect the initial wetting and spreading behaviour of the formulations. On human skin, the rapid evaporation and absorption of the aqueous phase are expected to progressively shift the surface properties toward a more hydrophobic character. While absolute contact angle values cannot be directly extrapolated, the relative trends observed between formulations remain meaningful for comparing their early interfacial behaviour and their initial sensory perception.

Figure 2 shows that all creams increase skin glossiness both immediately and 5 min after application. Initial measurements undoubtedly indicate that the presence of HNES enhances gloss properties (example C_C8C12: 16.4 ± 1.6 UA) compared with the MCT used in the control cream (11.9 ± 0.3 UA). However, no significant differences were observed between tested HNES at this time point. After 5 min, glossiness levels decrease, likely due to the evaporation of water contained in the creams, as the Biody® device does not simulate the human skin absorption capacity. In addition, both C_C8C12 and C_C12M emulsions do not show significant differences from the control (respectively 10.4 ± 1.2 UA; 10.5 ± 1.5 UA; 9.7 ± 2.1 UA), thus indicating that the HNES in these formulations provide glossiness levels like those MCT after 5 min of application. In contrast, both C_C8P_5:1_ (11.1 ± 1.6 UA) and C_C8P_1:2_ (11.2 ± 1.7 UA) creams show statistically significant differences from the control after 5 min of application.

**FIGURE 2:**
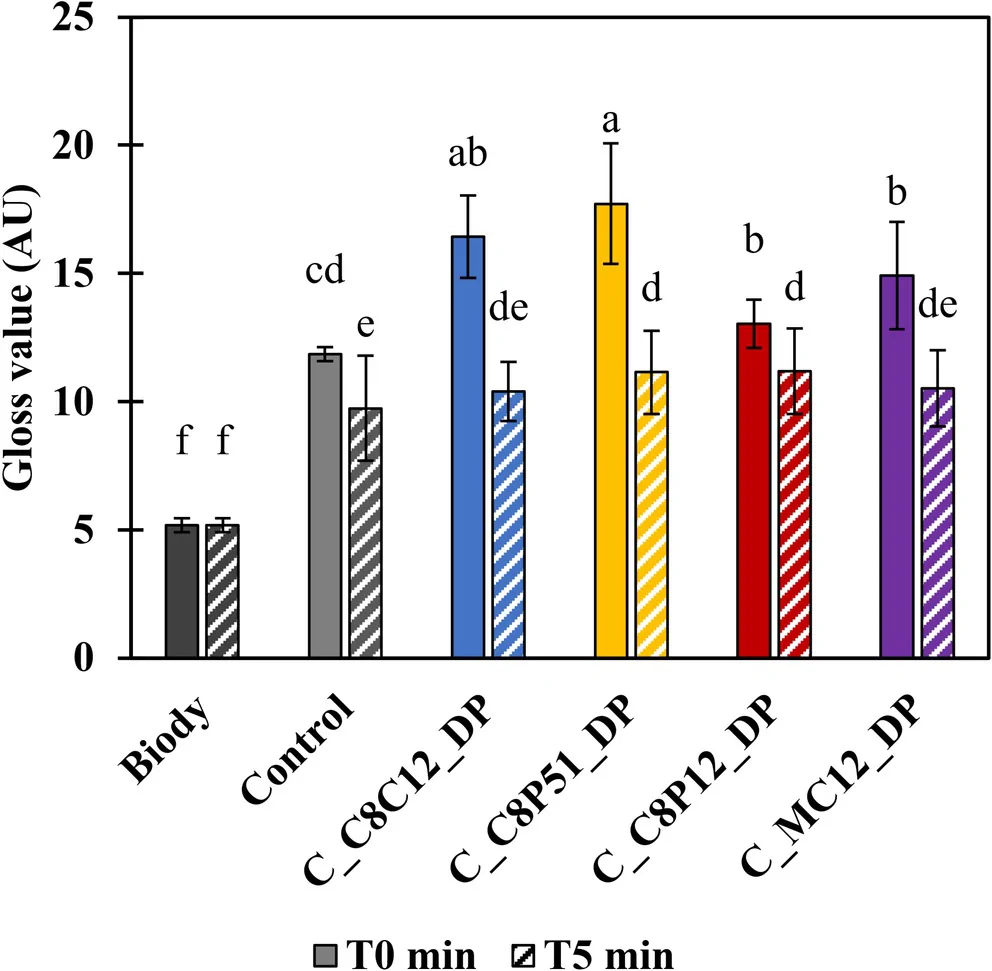
Mean value and standard deviation for glossiness and evolution of glossiness between application and 5 min after application. C_C8C12 (blue), C_C8P5:1 (yellow), C_C8P1:2 (red), C_C12M (violet). (a–e) Means sharing common superscripts are non‐significantly different as tested by Tukey's test (*p* > 0.01).

## DISCUSSION

The development of eutectic solvents as a green alternative to dissolve compounds of interest led researchers to interest themselves in the incorporation of this new type of ingredient in complex formulations. Gels and eutectogels were the first systems to be explored [6, 41, 42]. Emulsions, even if they are very common in food, pharmacy, and cosmetic fields, are poorly studied. The first works were dedicated to the incorporation of hydrophilic NES in the internal or external phase of an emulsion or cream [7, 43, 44, 45, 46]. Concerning hydrophobic eutectic systems, they were incorporated at a small concentration in anhydrous formulas or emulsions [43, 47].

A few studies describe the use of hydrophobic or hydrophilic eutectic solvents as a whole phase (internal or external) of an emulsified system. Nevertheless, those systems are rather complex, like microemulsions [48, 49], nanoemulsions [50, 51, 52, 53] or Pickering emulsions [46, 54] or use high energy methods and/or surfactants like polysorbates or sodium dodecyl sulphate, which are excluded from the COSMOS standard [50, 52, 53, 55]. The present study is the first one evaluating the feasibility of preparing a COSMOS‐certifiable cream where the internal phase is entirely made of a HNES and prepared with low‐energy‐consuming equipment. The closest published study is that of Xu and coll., who prepared emulsions whose internal phase is composed of a HNES made of linoleic acid, an unsaturated fatty acid prone to oxidation, and lecithin, a natural surfactant. The internal phase was composed of 2/3 of the HNES and 1/3 of pure retinol. The emulsion containing 30% wt. of this mix was prepared by high shear mixing followed by high pressure homogenization, leading, as expected, to a nanoemulsion [56]. The present study is then the first of its kind, comparing the potential of common HNES to be used as the internal phase of model cream.

The three HNES families studied exhibit distinct profiles in terms of functional properties and formulation behaviour.

The formulations C_C8M, C_C12M, and C_MO share characteristics related to the presence of menthol but exhibit notable differences. C_C8M and C_MO are the least stable, showing phase separation before D30. Their initial viscosity is high (102.8 and 104.9 Pa·s at 0.1 s^−1^, respectively), reflecting a dense structure but poor compatibility with the emulsifying system. In contrast, C_C12M, although less stable than fatty acid‐based creams, maintains sufficient physical integrity for evaluation: it displays the highest viscosity (215.6 Pa·s at 0.1 s^−1^) and a high flow threshold (32.7 Pa), indicating a highly resistant internal structure. From a sensory perspective, C_C12M provides acceptable lubrication but more pronounced stickiness (0.320 g·s), which may reduce application comfort. Overall, menthol incorporation tends to increase viscosity and rigidity in creams, but at the cost of compromised stability. These results are consistent with previous observations concerning the introduction of 10% of this type of HNES into a cream [47].

The formulations C_C8P_5:1_ and C_C8P_1:2_, both enriched with a more polar HNES, exhibit contrasting behaviours related to their 1,3 propanediol concentration. C_C8P_5:1_, with a higher proportion of C8, stands out for its better stability (droplet diameter increasing from 9.0 to 11.2 μm) and relatively constant pH (4.27–4.21), indicating good compatibility with the emulsifying system. Its intermediate viscosity (122.9 Pa·s at 0.1 s^−1^) provides a balanced texture, combined with excellent sensory properties: high lubrication and low stickiness (0.142 g·s). Conversely, C_C8P_1:2_, where the diol predominates, shows reduced stability (droplets growing from 19.2 to 24.0 μm) and a marked pH drop (4.93–4.10), signs of a less robust structure. Its higher viscosity (147.2 Pa·s at 0.1 s^−1^) results in a thicker texture, though without fully compromising sensory comfort, as stickiness remains very low (0.083 g·s). Overall, a higher C8 ratio appears to favour stability and sensory performance, whereas an increased proportion of diol raises viscosity and weakens stability.

The C_C8C12 cream illustrates the benefits of fatty acid‐based HNES: it combines excellent stability (droplet diameter increasing only from 2.2 to 2.9 μm) with low viscosity (29.9 Pa·s at 0.1 s^−1^), ensuring a smooth, easy‐to‐spread texture. Its sensory profile is particularly favourable, featuring high lubrication, reduced tackiness (0.157 g·s), and a very low contact angle (12°), indicating that this HNES does not create a greasy film on the skin despite its composition. Compared with menthol or diol‐based creams, C_C8C12 stands out for its balance between stability, application comfort, and rheological properties, making it a formulation particularly suited for stable and pleasant‐to‐use cosmetic products.

Information concerning the interest of the HNES components appears from the analysis of the results. Fatty acids, known for their emollient and nourishing properties, promote skin suppleness and protection. HNES‐based creams containing them (e.g., C_C8C12) stand out for their excellent stability, low viscosity, and optimal sensory comfort (low stickiness, good spreadability). They represent ideal bases for stable and pleasant‐to‐apply cosmetic products. Menthol, valued for its refreshing and soothing effects, provides an immediate sensation of comfort. However, its incorporation into HNES leads to a significant increase in viscosity and marked instability of the creams, especially when combined with octanediol. Although some formulations such as C_C12M retain a robust internal structure, they present sensory risks (higher stickiness) and limited compatibility with emulsifiers.

1,3‐propanediol, usually used for its humectant and moisturizing properties, enhances water retention and cream texture. Formulations based on this diol show variable stability depending on the ratio: a higher proportion of C8 (C_C8P_5:1_) promotes stability and sensory comfort, whereas a ratio in favour of propanediol (C_C8P_1:2_) increases viscosity and weakens stability. Overall, these creams offer a good compromise between hydration and sensoriality but require precise ratio adjustments to avoid destabilization.

In summary, for stable and pleasant cosmetic formulations, fatty acid‐based HNES appear to be the most reliable, while menthol‐based HNES, although sensorially attractive, require optimization to limit stability issues. Diol‐based HNES may offer an interesting approach to improving hydration due to the humectant properties of diols, provided their impact on rheology and stability is carefully controlled.

## CONCLUSION

This study demonstrates that HNES can fully replace the conventional oil phase in cosmetic creams using standard cold processes. Among the systems tested, fatty acid‐based HNES provided the best balance of stability, rheology, and sensory performance, producing small, stable droplets and low‐viscosity creams with excellent spreadability and minimal stickiness. Menthol‐containing and diol‐rich systems offered structured textures and improved gloss but showed reduced long‐term stability, requiring further optimization.

Overall, HNES represent a viable, regulation‐compliant alternative for eco‐designed emulsions, supporting industrial scalability and COSMOS certification. Future work should focus on improving the stability of menthol and diol systems, assessing compatibility with actives and preservatives. Furthermore, after safety studies are performed on each system, consumer perception could be validated to accelerate market adoption.

## CONFLICT OF INTEREST STATEMENT

The authors declare no conflicts of interest.

## Data Availability

The data that support the findings of this study are available from the corresponding author upon reasonable request.
